# The Proton-Sensing GPR4 Receptor Regulates Paracellular Gap Formation and Permeability of Vascular Endothelial Cells

**DOI:** 10.1016/j.isci.2020.100848

**Published:** 2020-01-17

**Authors:** Elizabeth A. Krewson, Edward J. Sanderlin, Mona A. Marie, Shayan Nik Akhtar, Juraj Velcicky, Pius Loetscher, Li V. Yang

**Affiliations:** 1 Department of Anatomy and Cell Biology, East Carolina University, Greenville, NC 27834, USA; 2 Department of Internal Medicine, Brody School of Medicine, East Carolina University, Greenville, NC 27834, USA; 3 Novartis Institutes for BioMedical Research, 4002 Basel, Switzerland

**Keywords:** Pathophysiology, Molecular Mechanism of Behavior, Specialized Functions of Cells

## Abstract

GPR4 is a pH-sensing G protein-coupled receptor highly expressed in vascular endothelial cells and can be activated by protons in the inflamed tissue microenvironment. Herein, we report that acidosis-induced GPR4 activation increases paracellular gap formation and permeability of vascular endothelial cells through the G_α12/13_/Rho GTPase signaling pathway. Evaluation of GPR4 in the inflammatory response using the acute hindlimb ischemia-reperfusion mouse model revealed that GPR4 mediates tissue edema, inflammatory exudate formation, endothelial adhesion molecule expression, and leukocyte infiltration in the inflamed tissue. Genetic knockout and pharmacologic inhibition of GPR4 alleviate tissue inflammation. These results suggest GPR4 is a pro-inflammatory receptor and could be targeted for therapeutic intervention.

## Highlights

• Acidosis/GPR4 regulates endothelial paracellular gap formation and permeability
• GPR4 exacerbates inflammation by increasing tissue edema and leukocyte infiltration
• Pharmacological inhibition of GPR4 reduces inflammatory responses

## Graphical Abstract

**Figure:**
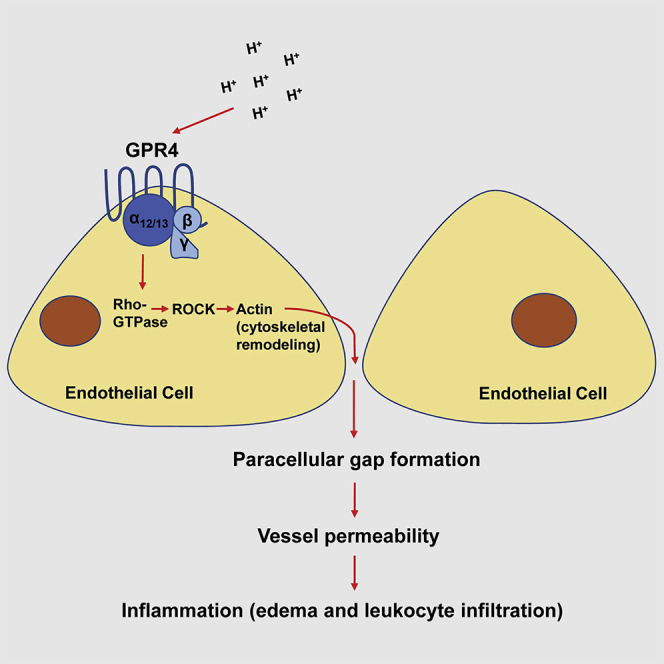

## Introduction

The endothelium is a dynamic barrier that can mediate the transvascular movement of fluids and immune cells between the peripheral blood and interstitial tissues. During active inflammation, the production of numerous inflammatory mediators within the inflammatory loci can increase endothelial gap formation and vascular permeability, which facilitate leukocyte infiltration and exudate formation in the inflamed tissues (Edlow and Sheldon, 1971, Muller, 2003, Pate et al., 2010). Accumulating evidence suggests that microenvironmental factors, such as acidic pH, can stimulate endothelial cell inflammation (Chen et al., 2011, Dong et al., 2013, Dong et al., 2017b, Okajima, 2013, Tobo et al., 2015). Inflammatory tissues are characteristically acidic, owing in part to hypoxia and increased glycolytic metabolism of cells and infiltrated leukocytes resulting in heightened proton production and accumulation. The acidic tissue microenvironment is associated with a wide range of inflammation-related disease states such as arthritis, inflammatory bowel disease, myocardial infarction, stroke, and limb ischemia. Previous reports note that local pH ranging from 6.0 to 7.0 is common in the microenvironments of inflamed tissues, solid tumors, and ischemic tissues (Huang and McNamara, 2004, Justus et al., 2013, Lardner, 2001, Siesjo et al., 1996). In ischemic disease, one study demonstrated that, within 50 min of coronary artery occlusion, local tissue extracellular pH decreased from 7.4 to 5.5 in domestic pigs (Hirche et al., 1980). Furthermore, in the tourniquet-induced rabbit limb ischemia model, local tissue pH decreased rapidly within 1 h and dropped from 7.30 to 6.36 during the 4-h course of limb ischemia (Hagberg, 1985). In summary, an acidic interstitial pH is an inflammatory microenvironmental factor in many pathological conditions and has been demonstrated to modulate tissue, blood vessel, and immune cell functions (Huang and McNamara, 2004, Justus et al., 2013, Lardner, 2001, Siesjo et al., 1996).

Cells can sense extracellular acidification through multiple molecular sensors such as acid-sensing ion channels (ASICs), transient receptor potential (TRP), and proton-sensing G protein-coupled receptors (GPCRs) (Holzer, 2009, Justus et al., 2013, Okajima, 2013, Sanderlin et al., 2015). GPR4 is a member of the proton-sensing GPCR family, which also includes GPR65 (TDAG8) and GPR68 (OGR1) (Justus et al., 2013, Ludwig et al., 2003, Okajima, 2013, Sanderlin et al., 2015, Yang et al., 2007). GPR4 is highly expressed in vascular endothelial cells (ECs) and has been shown to increase the expression of inflammatory cytokines, chemokines, adhesion molecules, and ER stress-related genes upon activation by acidic pH in ECs (Chen et al., 2011, Dong et al., 2013, Tobo et al., 2015). GPR4 has also been shown to potentiate inflammation *in vivo*. Recent studies found that the genetic deletion of GPR4 in mouse colitis models decreased the expression of endothelial adhesion molecules VCAM-1 and E-Selectin in the intestinal microvasculature, which was associated with reduced mucosal leukocyte infiltration and intestinal inflammation (Sanderlin et al., 2017, Wang et al., 2018). Furthermore, GPR4 was shown to increase tissue injury in a renal ischemia-reperfusion mouse model (Dong et al., 2017a).

Our current study focuses on the acidosis/GPR4-mediated endothelial paracellular gap formation and vessel permeability in the inflammatory response. Using genetic and pharmacological approaches, we demonstrate that activation of GPR4 by acidosis induces endothelial paracellular gap formation and permeability through the Gα_12/13_ signaling pathway. Furthermore, we demonstrate that the genetic deletion and pharmacological inhibition of GPR4 decrease blood vessel permeability, tissue edema, and leukocyte infiltration in the acute hindlimb ischemia-reperfusion mouse model. Our data suggest that GPR4 has a proinflammatory role in the regulation of the inflammatory response.

## Results

### Acidosis Promotes Paracellular Gap Formation in Primary Endothelial Cells

**Figure 1:**
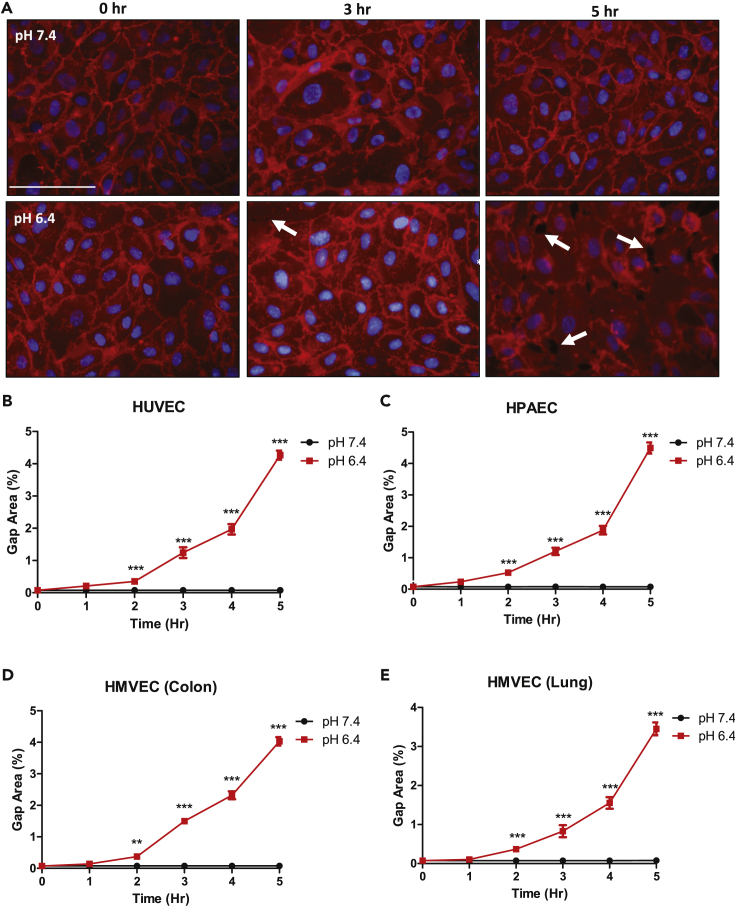
Acidosis Stimulates Paracellular Gap Formation in Primary Vascular Endothelial Cells (ECs) Plasma membrane staining and paracellular gap area quantitation of ECs treated for up to 5 h under physiological or acidic pH. Acidosis increases EC gap formation when compared with physiological pH treatment conditions. (A) Representative pictures of plasma membrane staining in human umbilical vein endothelial cells (HUVECs) at 0, 3, and 5 h treated under physiological or acidic pH. (B–E) Quantitative analysis of gap formation in (B) HUVECs, (C) human pulmonary artery endothelial cells (HPAECs), (D) human colon microvascular endothelial cells (HMVEC-Colon), and (E) human lung microvascular endothelial cells (HMVEC-Lung) over 5 h. All experiments were performed in triplicate and are representative of four experiments. Data at each time point are presented as mean ± SEM and analyzed for statistical significance between the pH 7.4 group and the pH 6.4 group using the unpaired t test where **p < 0.01 and ***p < 0.001. White arrows point to paracellular gaps. Scale bar, 100 μm.

Tissue acidosis commonly exists in inflammatory microenvironments (Dong et al., 2014b, Justus et al., 2013, Lardner, 2001, Okajima, 2013, Sanderlin et al., 2015). However, the involvement of acidosis in endothelial cell (EC) gap formation is largely unknown. Four primary vascular ECs were cultured to a confluent monolayer and were treated with either physiological pH 7.4 or acidic pH 6.4 for 5 h to assess acidosis-induced paracellular gap formation. Under physiological pH 7.4, all ECs maintained a cellular monolayer with no gap formation over the 5-h time course. However, under acidic pH 6.4 the cellular monolayers were disrupted and paracellular gap formation was observed (Figure 1A). The gap formation of EC monolayers was determined in human umbilical vein endothelial cells (HUVECs), human pulmonary artery ECs (HPAECs), human colon microvascular ECs (HMVECs-Colon), and human lung microvascular ECs (HMVECs-Lung) by calculating the total percent area of gaps in each field of view (Figures 1B–1E). ECs treated with acidic pH 6.4 for 5 h developed approximately 4%–5% gap area relative to the total area (Figures 1B–1E). No gaps, however, could be detected within physiological pH conditions.

### Acidosis Stimulates Endothelial Paracellular Gap Formation through the Proton-Sensing GPR4 Receptor

**Figure 2:**
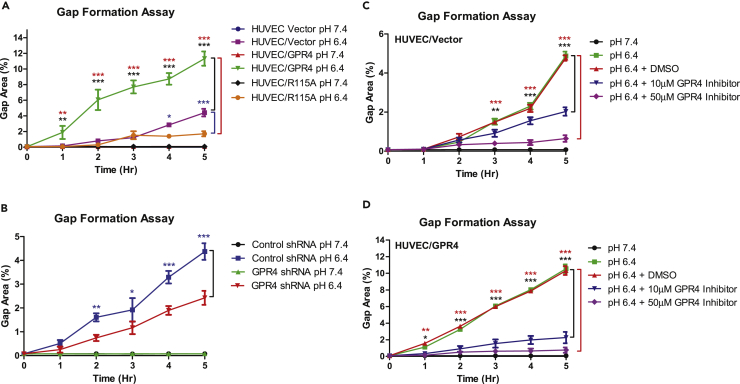
Activation of GPR4 by Acidosis Induces Paracellular Gap Formation in HUVECs (A–D) Quantitative analysis of gap formation utilizing genetic and pharmacological approaches to modulate GPR4 expression or activity. (A) HUVEC/vector, HUVEC/GPR4, and HUVEC/R115A cells. (B) HUVEC/control shRNA and HUVEC/GPR4 shRNA cells. (C) HUVEC/vector and (D) HUVEC/GPR4 cells treated with GPR4 inhibitor or vehicle. All cells were treated with physiological pH 7.4 or acidic pH 6.4 for 0, 1, 2, 3, 4, and 5 h and the percent of gap area was quantified. DMSO was used as vehicle control. All experiments were performed in duplicate or triplicate and are representative of three experiments. Data at each time point are presented as mean ± SEM and analyzed for statistical significance using the ANOVA where *p < 0.05, **p < 0.01, and ***p < 0.001. Black, blue, and red symbols indicate statistical analysis between groups indicated by bracket markers.

To determine the role of the pH-sensing receptor GPR4 in acidosis-induced EC gap formation, we used genetic and pharmacological approaches to modulate GPR4 expression and activity, respectively. HUVECs were stably transduced with either control (HUVEC/vector), GPR4 overexpression (HUVEC/GPR4), or GPR4 signaling-defective mutant (HUVEC/GPR4 R115A) overexpression constructs. GPR4 knockdown was achieved with transduction of GPR4 shRNA (HUVEC/GPR4 shRNA) and compared with control shRNA (HUVEC/control shRNA). As previously described (Chen et al., 2011, Dong et al., 2013), real-time RT-PCR analysis showed that GPR4 mRNA expression was increased by ∼14-fold in the overexpression HUVEC cells and decreased by >90% in the shRNA knockdown cells (Figure S1). HUVECs were evaluated under physiological pH 7.4 or acidic pH 6.4 for 5 h. Treatment with physiological pH 7.4 resulted in no observable gap formation. However, under acidic pH 6.4, HUVEC/vector cell monolayers developed ∼4% gap formation (Figure 2A). Overexpression of GPR4 significantly increased acidosis-induced gap formation by ∼2.5 fold (∼10%–11%) under acidic conditions when compared with HUVEC/vector. Conversely, HUVEC/GPR4 R115A mutant decreased the percentage of gap area when compared with HUVEC/vector (∼1.8% versus ∼4%, respectively). Furthermore, knockdown of GPR4 by shRNA decreased the percentage of gap area to ∼2.5% in acidic pH 6.4 conditions when compared with HUVEC/control shRNA (∼4.5%) (Figure 2B).

We next assessed the effects of a GPR4 inhibitor (EIDIP) on acidosis-induced paracellular gap formation in HUVEC/vector and HUVEC/GPR4 cells (Figures 2C and 2D). Pharmacological inhibition of GPR4 attenuated acidosis-induced gap formation. A dose-dependent decrease in the acidosis-induced gap development could be observed with increasing inhibitor concentrations during HUVEC/vector and HUVEC/GPR4 treatments when compared with vehicle (Figures 2C and 2D). The results indicate that acidosis-induced EC paracellular gap formation is dependent on GPR4 activation by acidic pH.

### GPR4-Mediated Gα_12/13_/Rho GTPase Signaling Is Involved in Endothelial Paracellular Gap Formation in Response to Acidosis

**Figure 3:**
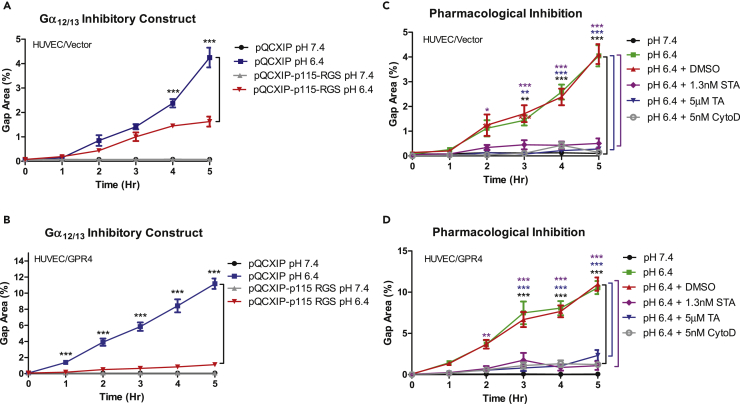
The Gα_12/13_/Rho GTPase Pathway Is Involved in Acidosis/GPR4-Mediated Paracellular Gap Formation in HUVECs (A–D) Quantitative analysis of GPR4/Gα_12/13_-mediated paracellular gap formation in HUVECs. GPR4-mediated paracellular gap formation is dependent, at least in part, on the Gα_12/13_/Rho GTPase/ROCK/MLCK/actin cytoskeleton pathway in response to acidic pH. HUVECs were treated with various inhibitors, including p115-RGS (an inhibitory construct of G_α12/13_), thiazovivin (a Rho-associated kinase ROCK inhibitor), staurosporine (a myosin light-chain kinase MLCK inhibitor), and cytochalasin D (an actin cytoskeleton inhibitor). The Gα_12/13_ p115-RGS inhibitory construct in (A) HUVEC/vector and (B) HUVEC/GPR4 cells. Pharmacological inhibition of the Gα_12/13_/Rho GTPase/ROCK/MLCK/actin cytoskeleton pathway using 5 μM thiazovivin (TA), 1.3 nM staurosporine (STA), or 5 nM cytochalasin D (CytoD) in (C) HUVEC/vector or (D) HUVEC/GPR4 cells. HUVECs were treated with either physiological pH 7.4 or acidic pH 6.4 for up to 5 h. All experiments were performed in duplicate and are representative of at least three experiments. Data at each time point are presented as mean ± SEM and analyzed for statistical significance using ANOVA where *p < 0.05, **p < 0.01, and ***p < 0.001. Black, blue, and purple symbols indicate statistical analysis between groups indicated by bracket markers.

Previous studies demonstrate that the Gα_12/13_/Rho GTPase pathway can regulate cytoskeletal dynamics, endothelial gap formation, and endothelial permeability (Thennes and Mehta, 2012, van Buul and Hordijk, 2004). In line with these observations, GPR4 can couple to Gα_12/13_ and Rho GTPase when expressed in cancer cell lines (Castellone et al., 2011, Justus and Yang, 2015). For this reason, we investigated the role of the GPR4/Gα_12/13_/Rho GTPase pathway in acidosis-induced EC gap formation. The p115 RGS Gα_12/13_ inhibitory construct (Kozasa et al., 1998, Yang et al., 2005) was stably transduced into HUVEC/vector and HUVEC/GPR4 cells. We next performed the gap formation assay under physiological and acidic pH conditions for 5 h. HUVEC/p115 RGS cells treated with acidic pH had significantly reduced gap formation when compared with the vector control (Figures 3A and 3B). Furthermore, thiazovivin and staurosporine, two chemical inhibitors for Gα_12/13_ downstream effectors Rho-associated kinase (ROCK) and myosin light-chain kinase (MLCK), respectively (Justus and Yang, 2015), were used in HUVEC/vector and HUVEC/GPR4 cells under acidic conditions. Thiazovivin significantly decreased gap formation percentage in HUVEC/vector and HUVEC/GPR4 cells when compared with the vehicle controls under acidic pH. Staurosporine nearly abolished acidosis-induced gap formation in HUVEC/vector and HUVEC/GPR4 cells (Figures 3C and 3D). Collectively, the results suggest that acidosis-induced EC gap formation relies, at least in part, on the GPR4/Gα_12/13_ pathway. Additionally, our results demonstrated that acidosis also induced F-actin stress fiber formation and decreased VE-cadherin expression at the site of paracellular gaps in ECs (Figures S2 and S3). To assess the role of actin cytoskeleton in acidosis/GPR4-mediated EC gap formation, HUVECs were treated with an actin cytoskeleton inhibitor cytochalasin D (CytoD). Cytochalasin D significantly reduced acidosis-induced gap formation in HUVEC/vector and HUVEC/GPR4 cells (Figures 3C and 3D). Moreover, p115 RGS, thiazovivin, staurosporine, and cytochalasin D substantially decreased F-actin stress fiber formation in HUVECs (Figure S4). Taken together, these results suggest that the Gα_12/13_/Rho/ROCK/MLCK/actin cytoskeleton pathway is important for acidosis/GPR4-induced endothelial paracellular gap formation.

### Acidosis Increases Endothelial Cell Permeability through GPR4

**Figure 4:**
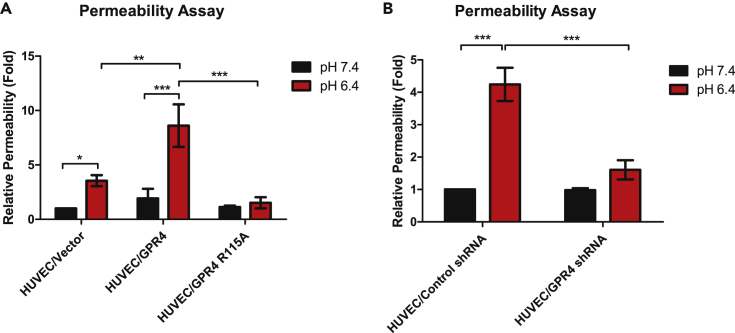
GPR4 Activation by Acidosis Increases Cellular Permeability in HUVECs (A and B) Quantitative analysis of cellular permeability *in vitro*. GPR4 activation by acidic pH increases cellular permeability using the FITC-dextran cellular permeability assay with HUVECs. Relative fold increase of cellular permeability in (A) HUVEC/vector, HUVEC/GPR4, and HUVEC/R115A cells and (B) HUVEC/control shRNA and HUVEC/GPR4 shRNA cells. Cells were treated for 5 h with either physiological pH 7.4 or acidic pH 6.4. All experiments were performed in duplicate and are representative of at least three experiments. Data are presented as mean ± SEM and analyzed for statistical significance using ANOVA where *p < 0.05, **p < 0.01, and ***p < 0.001.

We next assessed if GPR4-dependent paracellular gap formation can functionally result in increased endothelial permeability. Endothelial permeability was assessed by the fluorescein isothiocyanate conjugated-dextran (FITC-dextran) permeability assay whereby diffusion of FITC-dextran through the endothelial monolayer from the upper to lower chamber of the Transwell insert was assessed. Cells were treated with physiological pH 7.4 or acidic pH 6.4 for 5 h followed by the addition of FITC-dextran. Acidic pH treatment significantly increased FITC-dextran permeability in HUVEC/vector cells when compared with physiological pH. Moreover, when GPR4 is overexpressed and treated with acidic pH there was a further increase in acidosis-induced FITC-dextran when compared with HUVEC/vector cells. When GPR4 expression is knocked down or signaling is defective (R115A mutant) (Chen et al., 2011), FITC-dextran permeability was significantly decreased compared with controls under acidic conditions (Figure 4). These results suggest that acidosis increases EC permeability through GPR4.

### Genetic Deletion of GPR4 Reduces Tissue Edema and Inflammation in the Acute Hindlimb Ischemia-Reperfusion Mouse Model

**Figure 5:**
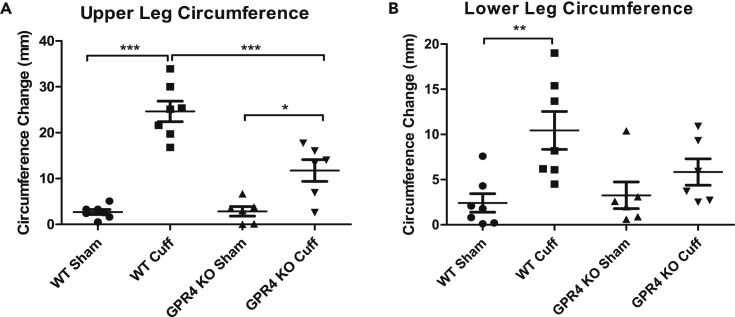
GPR4 Deficiency Reduces Tissue Edema in the Inflammatory Hindlimb Ischemia-Reperfusion (IR) Mouse Model (A and B) Upper and lower leg circumferences in wild-type (WT, N = 7) and GPR4 knockout (GPR4 KO, N = 6) mice were measured before and after IR. The change of leg circumference was calculated (value after IR – value before IR) and used as an indicator for tissue edema. GPR4 deficiency reduces the change of upper and lower leg circumference when compared with WT mice in the IR mouse model. Quantitative analysis of (A) upper and (B) lower leg circumference changes. Data are presented as mean ± SEM and analyzed for statistical significance using ANOVA where *p < 0.05, **p < 0.01, and ***p < 0.001.

Next, we assessed the functional role of GPR4 in a tourniquet cuff-based acute hindlimb ischemia-reperfusion mouse model (Bonheur et al., 2004). This mouse model can cause severe inflammation resulting in increased vessel permeability, tissue edema, and leukocyte infiltration in the affected tissue. The ischemia-reperfusion associated inflammation was induced in wild-type (WT) and GPR4 knockout (KO) mice. Consistent with previous report (Yang et al., 2007), the absence of GPR4 mRNA expression in KO mouse tissues was confirmed by RT-PCR (Figure S5). The sham and cuff limbs were measured for circumference differences between pre- and post-procedure measurements. GPR4 KO mice had less observable tissue edema in the tourniquet-affected limb following the ischemia and reperfusion event when compared with WT mice (Figure 5).

**Figure 6:**
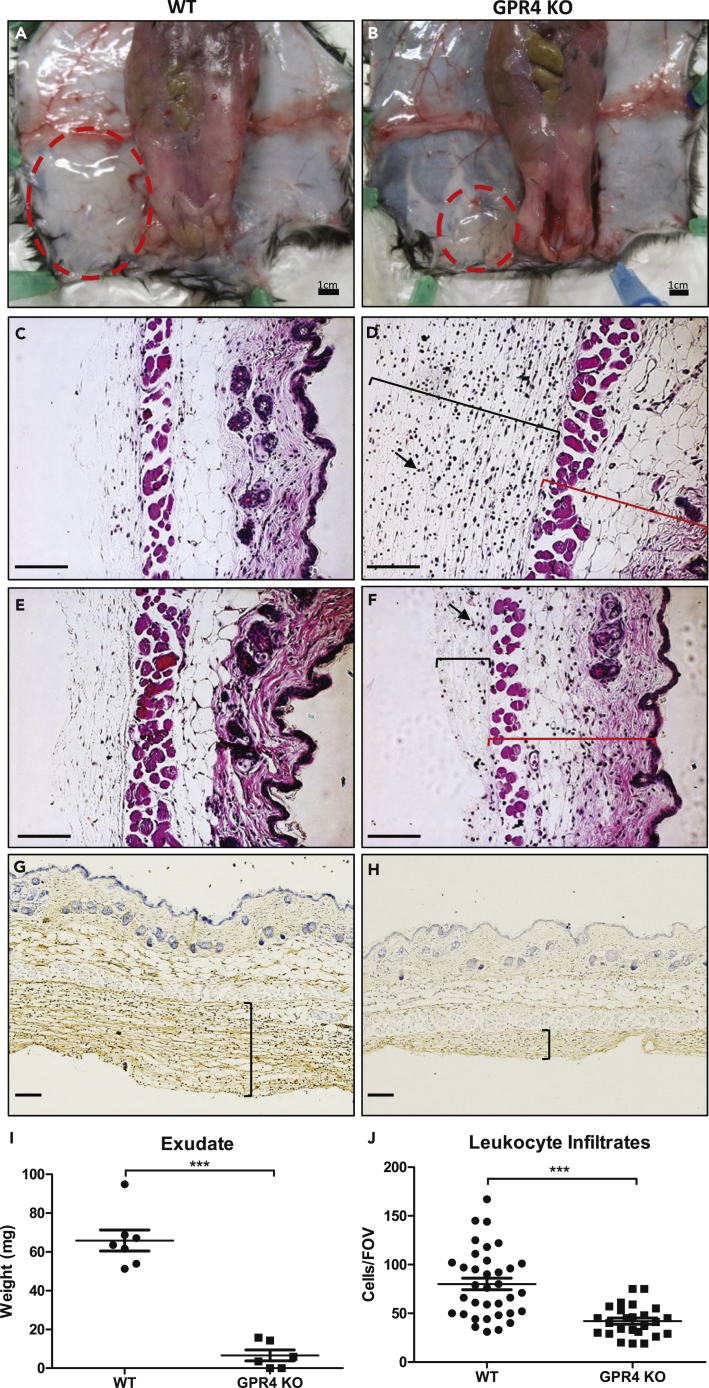
GPR4 Deficiency Reduces Inflammatory Exudate Production and Leukocyte Infiltration in the Ischemic Hindlimb Tissues (A–J) Inflammatory exudate measurements and leukocyte infiltration quantification in WT and GPR4 KO mice following the ischemia-reperfusion of the hindlimb. Genetic deletion of GPR4 reduces inflammatory exudate production and leukocyte infiltration into the inflammatory exudate. Gross representative images of observable exudate formation in (A) WT and (B) GPR4 KO mice in the interstitial space between the skin and muscle of the ischemic hindlimb. Representative images of H&E staining of (C) WT sham, (D) WT cuff, (E) GPR4 KO sham, and (F) GPR4 KO cuff inflammatory exudate sections. Black brackets indicate exudate distribution. Arrows indicate infiltrated leukocytes in the inflammatory exudate. Red brackets indicate skin tissues. Representative images of mouse plasma IgG protein in (G) WT cuff and (H) GPR4 KO cuff sections. IgG protein can be visualized as brown signal. The plasma protein IgG is used as a marker to indicate blood vessel permeability. Quantitative analysis of (I) exudate weight and (J) leukocyte infiltration from multiple fields of view (FOV). 10x and 20x microscope objectives used. Scale bar indicates 100 μm. Data analyzed from seven WT and six GPR4 KO mice. Data are presented as mean ± SEM and analyzed for statistical significance using the unpaired two-tailed t test where ***p < 0.001.

Inflammatory exudates in the interstitial space between the skin and the limb muscle/body peritoneum on the tourniquet subjected side were collected and measured followed by histology (Figure 6). GPR4 KO mice had reduced inflammatory exudate when compared with the WT mice (∼6 versus ∼65 mg, respectively) (Figures 6A, 6B, and 6I). Furthermore, histological analysis of the inflammatory exudates revealed that GPR4 KO mice had reduced leukocyte infiltrates when compared with WT (Figures 6D, 6F, and 6J). No red blood cells were observed in the exudate, suggesting that the exudate is due to increased vascular permeability but not vessel hemorrhaging (Figures 6A–6F). To further assess the GPR4-mediated vessel permeability, we performed immunohistochemistry for plasma protein immunoglobulin G (IgG) and detected IgG within inflammatory exudates (Figures 6G and 6H), indicating an increased vascular permeability to plasma protein. Furthermore, less plasma IgG could be observed in the GPR4 KO cuff-affected tissues compared with WT cuff-affected tissues indicating reduced endothelial cell permeability in the KO (Figures 6G and 6H). We also performed immunohistochemistry of CD31 (a pan-endothelial marker) to assess blood vessel density in the hindlimb dermis and hypodermis tissues. No significant difference in blood vessel density was observed between WT and GPR4 KO or between sham and cuff-affected dermis and hypodermis tissues within the 24-h ischemia and reperfusion (Figure S6).

**Figure 7:**
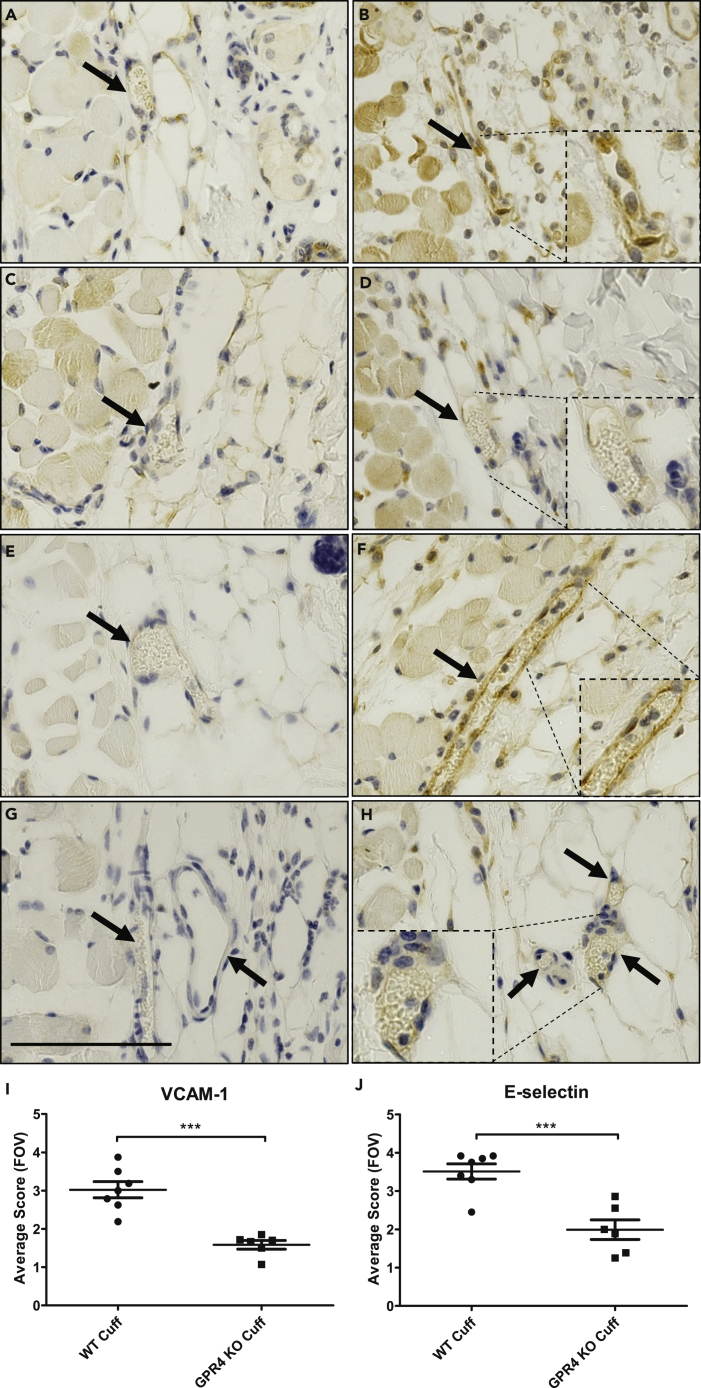
Immunohistochemistry of VCAM-1 and E-Selectin Protein Expression in the Loose Connective Dermal Tissue of WT and GPR4 KO Mice (A–J) Immunohistochemical analysis of adhesion molecules VCAM-1 and E-selectin protein expression in vascular endothelial cells within mouse dermal tissue sections of sham and cuff affected hindlimbs. GPR4-deficient mice had reduced expression of VCAM-1 and E-selectin in vascular endothelial cells in cuff-affected tissues when compared with WT. Representative pictures of VCAM-1 signal in (A) WT sham, (B) WT cuff, (C) GPR4 KO sham, and (D) GPR4 KO cuff tissues. Representative pictures of E-selectin expression can be visualized in (E) WT sham, (F) WT cuff, (G) GPR4 KO sham, and (H) GPR4 KO cuff tissues. Quantitative scoring analysis of (I) VCAM-1 and (J) E-selectin protein expression in vascular endothelial cells in WT and GPR4 KO cuff-affected dermal tissues. Pictures taken with 63x objective. Scale bar indicates 100 μm. Arrows indicate blood vessels. Data are presented as mean ± SEM and were analyzed for statistical significance using the unpaired two-tailed t test where ***p < 0.001.

To provide a molecular explanation for GPR4-mediated inflammation within vascular endothelial cells, we performed immunohistochemistry to examine the expression of VCAM-1 and E-selectin within the endothelium of the cuff and sham-affected dermis and hypodermis tissues. Overall, VCAM-1 and E-selectin protein expression was increased within the cuff-affected limb vasculature when compared with sham (Figure 7). Immunohistochemical analysis of VCAM-1 revealed expression on a variety of cell types, such as skeletal muscle, fibroblast, and vascular endothelial cells, which is consistent with the previous literature (Epperly et al., 2002, Ulyanova et al., 2005). Interestingly, we observed a decrease of VCAM-1 protein expression on vascular endothelial cells in GPR4 KO cuff-affected dermis and hypodermis of the limbs when compared with WT (Figures 7A–7D and 7I). E-selectin expression was observed on endothelial cells and fibroblasts as previously reported (Bajnok et al., 2017, Harashima et al., 2001, Vainer et al., 1998). Similar to VCAM-1 expression patterns, there is an observable decrease in endothelial E-selectin protein expression in GPR4 KO cuff-affected dermis and hypodermis of the limbs when compared with WT (Figures 7E–7H and 7J).

### Pharmacological Inhibition of GPR4 Reduces Tissue Edema, Leukocyte Infiltration, and Vascular Permeability in the Hindlimb Ischemia-Reperfusion Mouse Model

**Figure 8:**
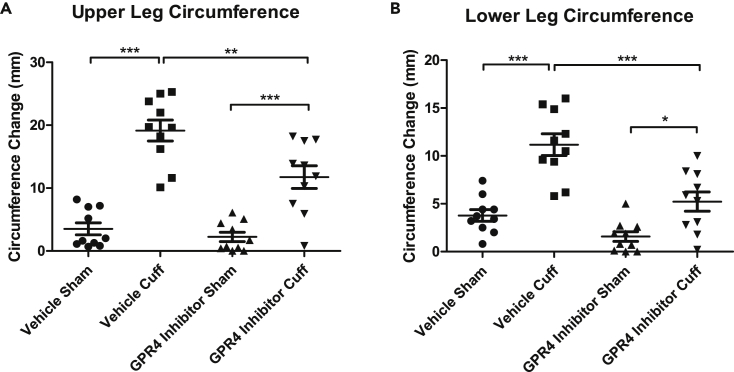
Pharmacological Inhibition of GPR4 Reduces Tissue Edema in the Inflammatory Hindlimb Ischemia-Reperfusion (IR) Mouse Model (A and B) Upper and lower leg circumference changes following IR in wild-type (WT) mice treated with either vehicle or GPR4 antagonist 13. GPR4 antagonist 13 reduces the changes of upper and lower leg circumference when compared with vehicle control in the IR mouse model. Quantitative analysis of (A) upper and (B) lower leg circumference changes, respectively. N = 10 vehicle- and N = 10 GPR4 antagonist 13-treated mice. Data are presented as mean ± SEM and were analyzed for statistical significance using the ANOVA where *p < 0.05, **p < 0.01, and ***p < 0.001.

**Figure 9:**
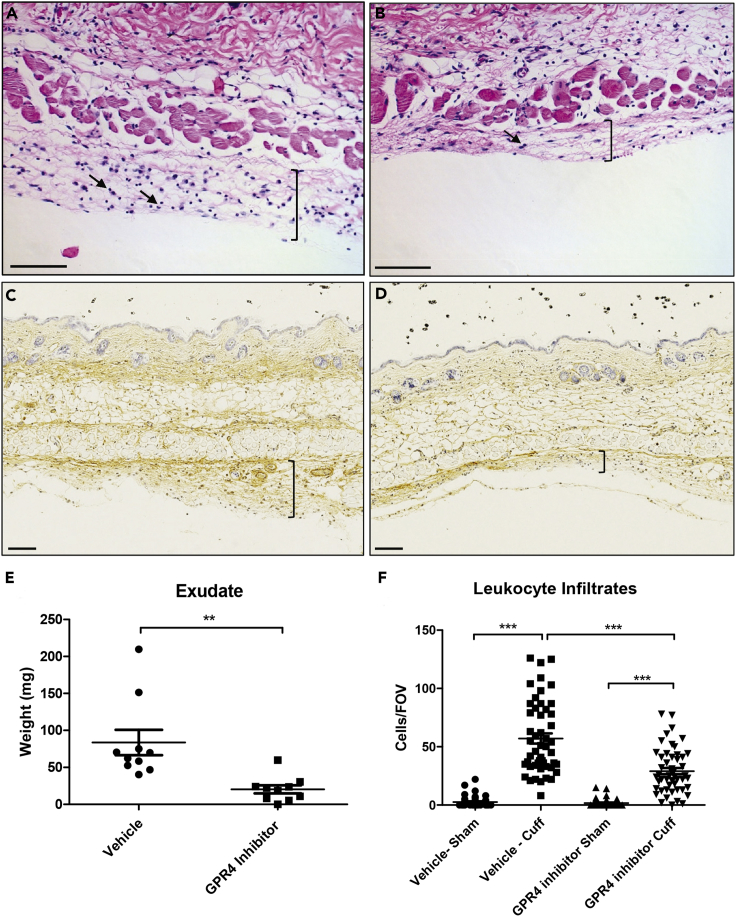
GPR4 Antagonist 13 Reduces Inflammatory Exudate Production and Leukocyte Infiltration in the Ischemic Hindlimb Tissues (A–F) Inflammatory exudate measurements and leukocyte infiltration quantification in WT mice provided with vehicle or GPR4 antagonist 13 in the hindlimb ischemia-reperfusion mouse model. GPR4 antagonist 13 reduces inflammatory exudate formation and leukocyte infiltration in the exudate. Representative images of H&E staining of (A) WT vehicle cuff and (B) WT GPR4 antagonist 13 cuff skin tissue sections. Immunohistochemical staining of mouse plasma IgG protein in (C) WT vehicle cuff and (D) WT GPR4 antagonist 13 cuff inflammatory exudate sections. IgG protein can be visualized as brown signal. (E) Total exudate formation and (F) leukocyte infiltration from multiple fields of view (FOVs) when compared with vehicle control following ischemia-reperfusion. Black brackets indicate exudate distribution. Arrows indicate infiltrated leukocytes. 10x and 20x microscope objectives used. Scale bars indicate 100 μm. Data analyzed from 10 vehicle- and 10 GPR4 inhibitor-treated mice. Data are presented as mean ± SEM and were analyzed for statistical significance using the unpaired two-tailed t test or ANOVA where **p < 0.01 and ***p < 0.001.

**Figure 10:**
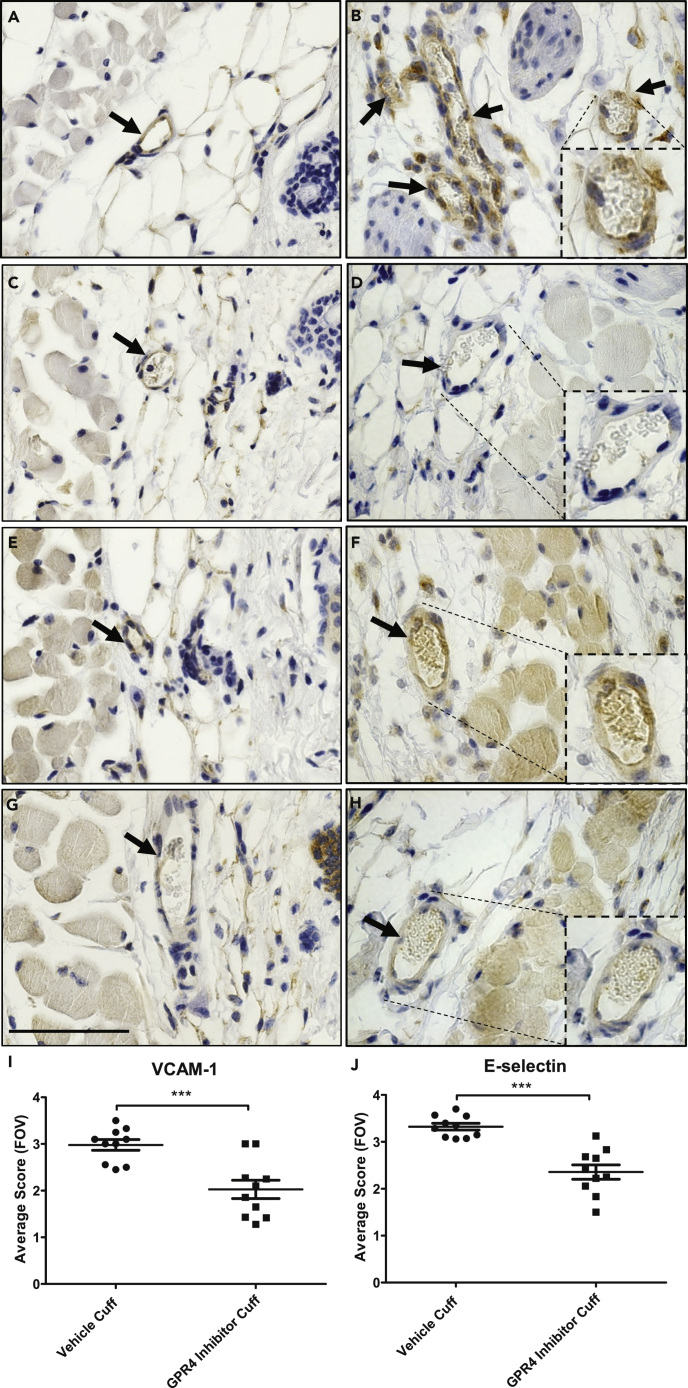
Immunohistochemistry of VCAM-1 and E-Selectin Protein Expression in the Loose Connective Dermal Tissue of Wild-Type Mice Treated with GPR4 Antagonist 13 or Vehicle (A–J) Immunohistochemical analysis of adhesion molecules VCAM-1 and E-selectin protein expression in vascular endothelial cells within mouse dermal tissue sections of sham and cuff-affected hindlimbs. WT mice treated with GPR4 antagonist 13 had reduced expression of VCAM-1 and E-selectin in vascular endothelial cells in cuff-affected tissues when compared with vehicle control. Representative pictures of VCAM-1 signal in (A) WT vehicle sham, (B) WT vehicle cuff, (C) WT GPR4 antagonist 13 sham, and (D) WT GPR4 antagonist 13 cuff dermal connective tissues. Representative pictures of E-selectin expression can be visualized in (E) WT vehicle sham, (F) WT vehicle cuff, (G) WT GPR4 antagonist 13 sham, and (H) WT GPR4 antagonist 13 cuff dermal tissues. Quantitative scoring analysis of (I) VCAM-1 and (J) E-selectin protein expression in vascular endothelial cells in WT vehicle and WT GPR4 antagonist 13 cuff-affected dermal tissues. Pictures taken with 63x microscope objective. Scale bar indicates 100 μm. Arrows indicate blood vessels. Data are presented as mean ± SEM and were analyzed for statistical significance using the unpaired two-tailed t test where ***p < 0.001.

To further assess the role of GPR4, we incorporated the use of a highly potent and selective GPR4 antagonist (referred to as GPR4 antagonist 13) (Velcicky et al., 2017) within the acute hindlimb ischemia-reperfusion mouse model. Our results demonstrated that the GPR4 antagonist 13 significantly decreased upper and lower limb tissue edema measured by the circumference of the leg compared with limbs of the vehicle control (Figure 8). In addition, there was significantly less leukocyte infiltration within the inflammatory exudate in the GPR4 antagonist 13-treated mice when compared with the vehicle-treated mice (Figures 9A, 9B, and 9F). Furthermore, the exudate weight was also decreased in GPR4 antagonist 13-treated mice when compared with vehicle (Figure 9E). Additionally, vascular permeability was assessed and there was a reduction in plasma IgG diffusion in the exudate by the treatment of the GPR4 antagonist 13 when compared with vehicle treatment (Figures 9C and 9D). GPR4 antagonist 13 treatment also reduced the level of an inflammatory marker, C-reactive protein (CRP), in the mouse serum (Figure S7). To further examine the role of GPR4 within ECs, we performed immunohistochemistry to analyze the expression of VCAM-1 and E-selectin within the endothelium of the cuff-affected dermis and hypodermis tissues. We observed that ECs in the GPR4 antagonist 13-treated mice had reduced VCAM-1 and E-selectin protein expression compared with the vehicle-treated mice (Figure 10). Taken together, these data suggest that GPR4 can mediate inflammation, vessel permeability, and leukocyte infiltration into inflamed tissues.

## Discussion

This study demonstrates that activation of GPR4 by acidic pH can stimulate endothelial inflammation through the mediation of paracellular gap formation and permeability by the Gα_12/13_ pathway. We also evaluate the functional role of GPR4 in the inflammatory response within the hindlimb ischemia-reperfusion mouse model using GPR4-null mice and a selective GPR4 antagonist (Velcicky et al., 2017). These data suggest that both the genetic knockout and pharmacological inhibition of GPR4 reduce the inflammatory response in the ischemia-reperfusion mouse model. These results indicate GPR4 could be a valuable therapeutic target for the remediation of inflammatory disease states.

The inflamed tissue microenvironment has been characterized by a loss of pH homeostasis. Acidosis is a microenvironmental stress factor in which both stromal and infiltrated immune cells exist and that alters cellular function (Lardner, 2001, Okajima, 2013, Sanderlin et al., 2015). Interestingly, local acidosis has been described as a danger signal within ischemic and inflamed tissues that can promote inflammation (Rajamaki et al., 2013). However, the way by which cells sense the altered acidic microenvironment and subsequently alter the inflammatory response has only recently been investigated. Cells can employ distinct acid-sensing mechanisms such as TRPs, ASICs, and proton-sensing GPCRs (Holzer, 2009, Okajima, 2013, Sanderlin et al., 2015, Wemmie et al., 2006). Proton-sensing GPCRs are activated by acidic pH through the protonation of several histidine residues on the extracellular domains of the receptors, which can induce conformational changes in the GPCRs for subsequent G protein activation and downstream signaling (Liu et al., 2010, Ludwig et al., 2003). The family of proton-sensing GPCRs include GPR4, GPR65 (TDAG8), and GPR68 (OGR1). GPR65 and GPR68 are predominately expressed in immune cells, whereas GPR4 is expressed in endothelial cells (Dong et al., 2014b, Justus et al., 2013, Okajima, 2013, Sanderlin et al., 2015). This family of receptors has only recently been implicated in the regulation of post-ischemic cellular responses and inflammation (Dong et al., 2014a, Ma et al., 2017, Matsuzaki et al., 2011, Russell et al., 2012, Sanderlin et al., 2015).

For example, GPR68 has been suggested to have a cardioprotective function by establishing a cellular protective border zone around the infarcted tissues (Russell et al., 2012). GPR65 expression was increased in rat brains and inhibited neuronal apoptosis and post-ischemic inflammatory responses following the middle cerebral artery occlusion and reperfusion event (Ma et al., 2017). Studies investigating the role of GPR4 under ischemic conditions are predominately focused on the kidney (Dong et al., 2014a). A recent study demonstrated that GPR4 deficiency improved renal ischemia-reperfusion injury clinical parameters such as survival rate, serum creatinine, and blood urea nitrogen levels. Furthermore, genetic deficiency of GPR4 deterred apoptosis by suppressing CHOP expression in the kidney (Dong et al., 2017a). This study is consistent with our previous report demonstrating CHOP expression is dependent on GPR4 activation in HUVECs (Dong et al., 2013, Dong et al., 2017b). However, these previous studies did not investigate the role of GPR4 in post-ischemic vessel permeability or inflammation.

Our current study reveals a functional role for GPR4 in governing endothelial paracellular gap formation and permeability in response to acidic stress through the Gα_12/13_ pathway. Using both genetic and pharmacological approaches, we demonstrated that acidosis-induced GPR4 activation significantly increases the paracellular gap formation and permeability when compared with physiological pH *in vitro*. The acidosis/GPR4-induced HUVEC paracellular gap formation is mediated through the Gα_12/13_/Rho GTPase/ROCK/actin cytoskeleton pathway. It has been previously reported that activation of GPR4 by acidosis can stimulate several downstream G protein pathways including G_12/13_, G_s_, and G_q_ (Liu et al., 2010, Ludwig et al., 2003). Future study is needed to elucidate the role of the complex signaling pathways of GPR4 in paracellular gap formation and other biological processes. Several potential mechanisms should be considered such as biased GPCR signaling and one G protein signaling pathway interacting with/overpowering another G protein signaling pathway. Other potential mechanisms, such as G protein-independent pathways and indirect effects due to cytokine/chemokine generation, should also be considered. Furthermore, a previous study reported that GPR4 can reduce endothelial cell barrier function via lysophosphatidylcholine (LPC) (Qiao et al., 2006). However, the LPC-GPR4 ligand binding interaction was unable to be confirmed (Zhu et al., 2001). Instead, GPR4 has been shown to function as a pH sensor (Ludwig et al., 2003, Yang et al., 2007). Altered endothelial cell permeability can lead to detrimental complications, such as tissue edema. Many diseases related to ischemia-induced inflammation are associated with increased vascular permeability such as stroke, myocardial infarction, sepsis, and cancer (Kumar et al., 2009). Our study suggests that GPR4 mediates endothelial paracellular gap formation and permeability in response to acidotic stress within the acidic inflamed microenvironment. For this reason, we further investigated the functional role of GPR4 in mediating acute inflammation using the hindlimb ischemia-reperfusion mouse model.

To investigate GPR4 in the ischemia-reperfusion model, we used the GPR4 KO mice for comparison with WT mice. Our data demonstrated that GPR4 KO mice had reduced parameters of acute inflammation such as tissue edema, inflammatory exudate formation, leukocyte infiltration, and EC adhesion molecule expression (VCAM-1 and E-selectin). These results suggest GPR4 mediates ischemia/reperfusion-induced inflammation most likely through vascular inflammatory programs such as increased vessel permeability and adhesion molecule expression. We and others have previously demonstrated that GPR4 regulates endothelial cell inflammation and ER stress responses (Chen et al., 2011, Dong et al., 2013, Dong et al., 2017b, Tobo et al., 2015). Activation of GPR4 in human endothelial cells by acidic pH increased numerous inflammatory cytokines, chemokines, cellular adhesion molecules, and ER stress-related genes (Dong et al., 2013, Dong et al., 2017b). Additionally, activation of GPR4 in HUVECs functionally mediated EC-leukocyte interactions, which are necessary for the leukocyte extravasation process in the inflammatory response (Chen et al., 2011, Dong et al., 2013). The role of GPR4 in the regulation of inflammation has recently been evaluated *in vivo* using the dextran sulfate sodium (DSS)-induced colitis mouse model (Sanderlin et al., 2017, Wang et al., 2018). GPR4 KO mice were protected from intestinal inflammation and had reduced adhesion molecule expression in intestinal microvascular endothelial cells when compared with WT mice (Sanderlin et al., 2017). These data suggest GPR4 potentiates inflammation likely through increased vascular endothelial cell inflammatory responses and are consistent with observations made in this current study and implicate GPR4 as a potential therapeutic target for the remediation of acute and chronic inflammation. It should also be noted that the GPR4 KO mice used in this study and other previous studies are global knockout with GPR4 deficiency in all tissue types (Dong et al., 2017a, Ludwig et al., 2003, Sanderlin et al., 2017, Wang et al., 2018, Yang et al., 2007). Although GPR4 is predominantly expressed in vascular endothelial cells, global knockout of GPR4 in other cell types might also have biological effects. Future research to generate endothelium-specific knockout mice will help further define the role of GPR4 in inflammatory responses and vascular biology.

Previous reports have indicated a group of imidazopyridine derivatives, found to selectively inhibit GPR4, can reduce both endothelial cell inflammation *in vitro* and tissue inflammation *in vivo* (Dong et al., 2013, Dong et al., 2017b, Fukuda et al., 2016, Miltz et al., 2017, Tobo et al., 2015). We previously demonstrated that GPR4 inhibitors can inhibit GPR4 activation in HUVECs following acidotic stimulation *in vitro*, which resulted in reduced expression of GPR4-mediated proinflammatory cytokines, chemokines, and cellular adhesion molecules (Dong et al., 2017b). Previous *in vivo* studies evaluated GPR4 inhibitors in myocardial infarction, arthritis, nociception, and angiogenesis mouse models and demonstrated that GPR4 inhibition reduced the disease severity when compared with vehicle control (Fukuda et al., 2016, Miltz et al., 2017). Recently, GPR4 antagonist 13, a pyrazolopyrimidine derivative was developed by Novartis Pharmaceuticals as the next generation of GPR4 inhibitors and found to be more selective for GPR4 inhibition and orally active (Velcicky et al., 2017). GPR4 antagonist 13 was also tested against other pH-sensing GPCRs, the H3 receptor, and hERG channel and demonstrated high selectivity for GPR4. The *in vivo* pharmacokinetics were also evaluated for GPR4 antagonist 13 and found to have good profiles of oral delivery and clearance. GPR4 antagonist 13 was found to effectively reduce arthritic inflammation, hyperalgesia, angiogenesis, and colitis (Sanderlin et al., 2019, Velcicky et al., 2017).

We sought to evaluate the anti-inflammatory effects of GPR4 antagonist 13 within the hindlimb ischemia-reperfusion mouse model. Notably, we observed similar effects of the pharmacological inhibition of GPR4 with GPR4 antagonist 13 when compared with the genetic knockout of GPR4. Our results demonstrated that GPR4 exacerbated post-ischemia/reperfusion tissue inflammation. GPR4 antagonist 13 administration resulted in a decrease in gross edema clinical parameters, inflammatory exudate formation, and leukocyte infiltration. Moreover, GPR4 antagonist 13 treatment reduced endothelial permeability as evidenced by a decrease in plasma IgG protein leakiness when compared with vehicle control. Proinflammatory modulators such as endothelial adhesion molecules (VCAM-1 and E-selectin) were also decreased by GPR4 antagonist 13 compared with vehicle.

Taken together, this study demonstrates that GPR4 activation by acidosis can induce endothelial paracellular gap formation and permeability *in vitro*. Furthermore, evaluation of the contribution of GPR4 in the hindlimb post-ischemia/reperfusion inflammatory response supports the role for GPR4 in vascular permeability and inflammation. Genetic knockout and pharmacological inhibition of GPR4 *in vivo* can decrease leukocyte infiltration and the expression of endothelial adhesion molecules VCAM-1 and E-selectin and can reduce vascular permeability as evidenced by attenuated plasma IgG leakiness into the subcutaneous connective tissues and exudate formation. The results suggest that inhibition of GPR4 can be exploited as a potential approach to alleviate inflammation and tissue edema.

### Limitations of the Study

This study focuses on the role of the proton-sensing receptor GPR4 in acidosis-mediated endothelial paracellular gap formation, permeability, and inflammatory response. In addition to the proton-sensing GPCRs, there are other types of acid sensors such as ASICs and TRPs (Holzer, 2009, Okajima, 2013, Sanderlin et al., 2015, Wemmie et al., 2006). It remains to be determined whether there are functional interactions between the proton-sensing GPCRs and other acid sensors in endothelial cell biology. Additionally, although this study and other preclinical studies demonstrate that the GPR4 antagonists exhibit anti-inflammatory, antinociceptive, and tissue-protective effects (Dong et al., 2013, Dong et al., 2017b, Fukuda et al., 2016, Miltz et al., 2017, Sanderlin et al., 2019, Tobo et al., 2015, Velcicky et al., 2017), the potential therapeutic effects of GPR4 antagonists in human patients remain to be evaluated.

## Methods

All methods can be found in the accompanying Transparent Methods supplemental file.

## Supplemental Information

Document S1. Transparent Methods and Figures S1–S7
