# Supplementary material for: The Proton-Sensing GPR4 Receptor Regulates Paracellular Gap Formation and Permeability of Vascular Endothelial Cells
Source: iScience. 2020 Jan 17;23(2):100848. doi: 10.1016/j.isci.2020.100848 (PMC6997876; doi:10.1016/j.isci.2020.100848)
Supplement: Document S1. Transparent Methods and Figures S1–S7 [file mmc1.pdf]

iScience, Volume *23*

Supplemental Information

The Proton-Sensing GPR4 Receptor Regulates Paracellular Gap Formation and Permeability of Vascular Endothelial Cells

Elizabeth A. Krewson, Edward J. Sanderlin, Mona A. Marie, Shayan Nik Akhtar, Juraj Velcicky, Pius Loetscher, and Li V. Yang

Supplemental Figures

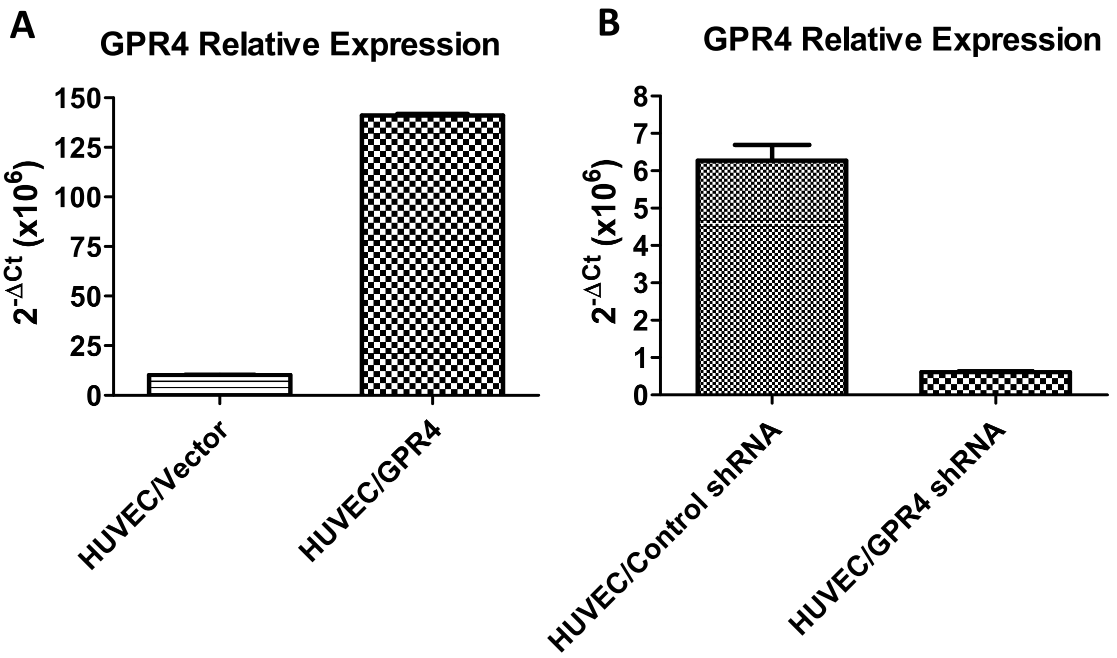

Figure S1. Expression of GPR4 mRNA in HUVECs. Related to Figure 2. (**A** and **B**) Real-time RT-PCR was performed to assess the expression of GPR4 mRNA in HUVEC/Vector, HUVEC/GPR4, HUVEC/Control shRNA and HUVEC/GPR4 shRNA cells. GPR4 was overexpressed ~14 fold in the HUVEC/GPR4 cells and knocked down >90% in the HUVEC/GPR4 shRNA cells when compared to the control HUVEC cells (**A** and **B**, respectively). Each cDNA sample was run in duplicate for the real-time RT-PCR analysis, and the 18S rRNA was used as the internal control for data normalization. The $2^{-\Delta Ct}$ method was used for data analysis.

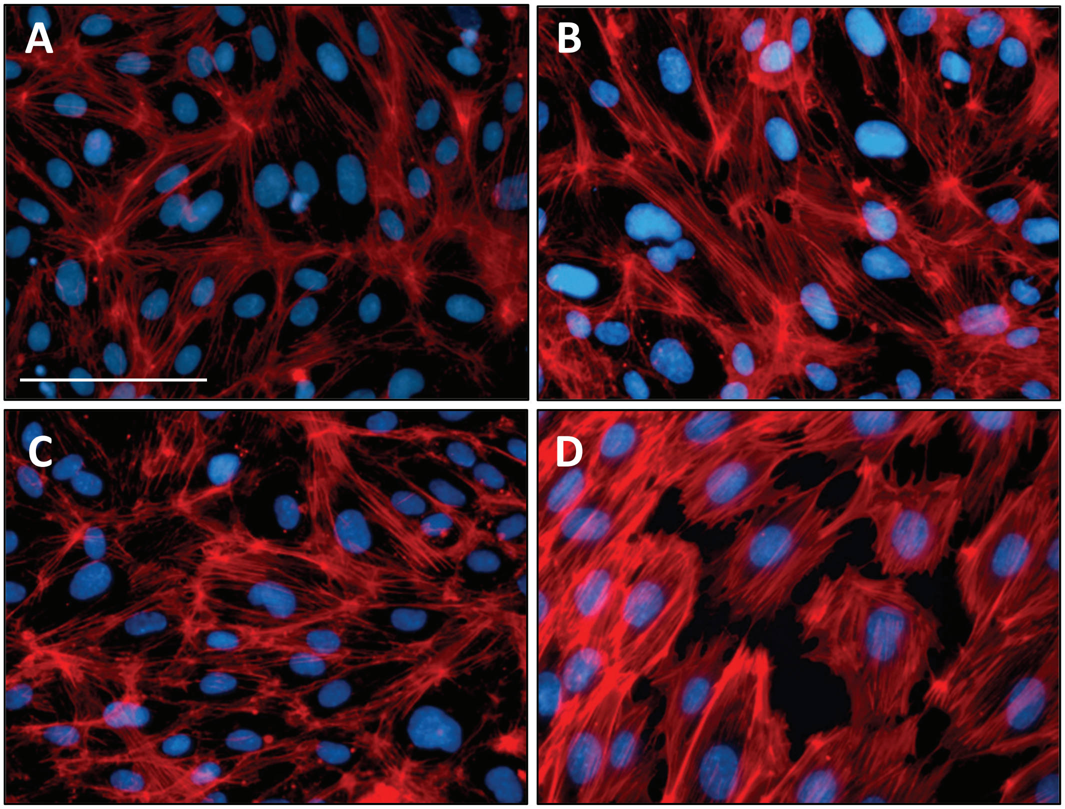

Figure S2. GPR4 activation by acidic pH induces actin stress fiber formation in HUVECs. Related to Figure 3. (**A-D**) Actin stress fiber formation in HUVECs treated with physiological or acidic pH. Activation of GPR4 by acidic pH 6.4 increases actin stress fibers when compared to physiological pH 7.4 in HUVECs. Representative images of F-actin labeled with rhodamine phalloidin in (**A**) HUVEC/vector pH 7.4, (**B**) HUVEC/vector pH 6.4, (**C**) HUVEC/GPR4 pH 7.4, and (**D**) HUVEC/GPR4 pH 6.4. HUVECs were treated with pH stimulation medium for 5 hours. Scale bar indicates 100µm.

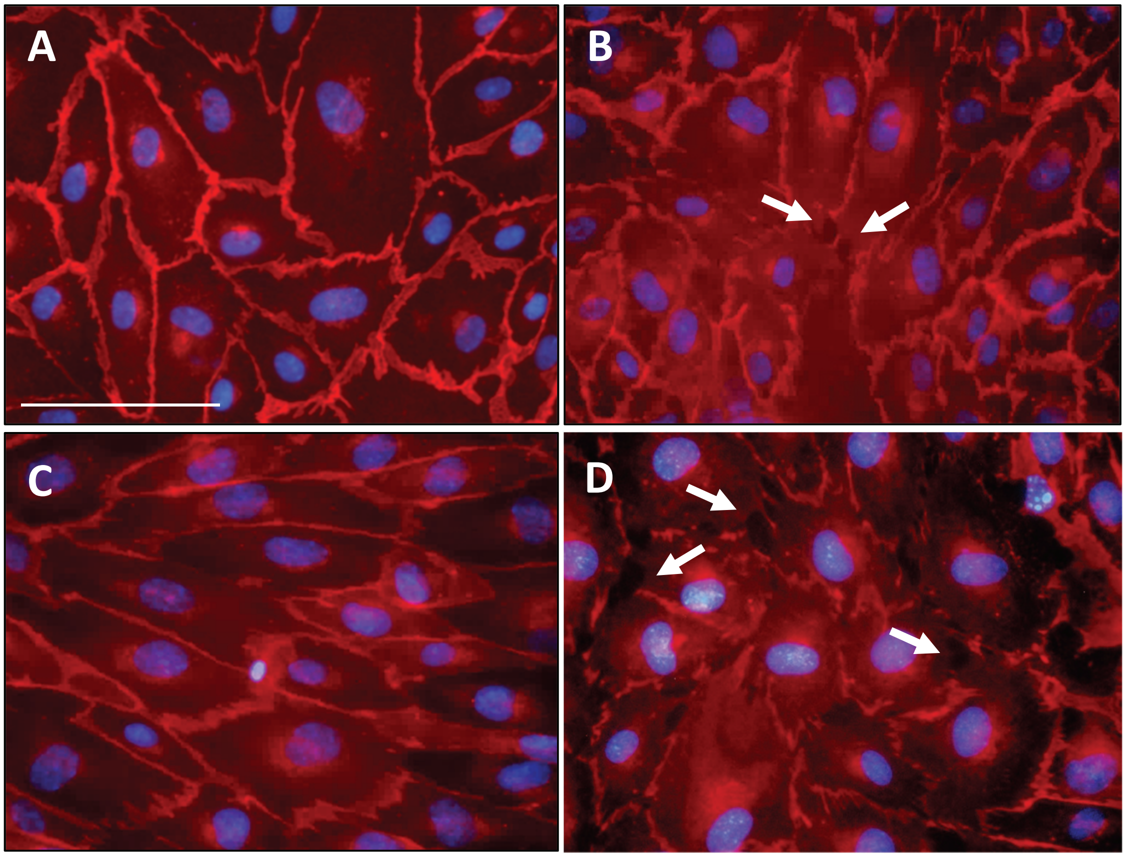

Figure S3. VE-Cadherin protein expression is reduced on endothelial paracellular gap borders. Related to Figure 3. (**A-D**) VE-Cadherin protein expression in HUVECs treated with physiological or acidic pH. Acidosis/GPR4-mediated endothelial paracellular gap areas have reduced VE-Cadherin expression along endothelial cell borders. Representative images of VE-Cadherin expression in (**A**) HUVEC/vector pH 7.4, (**B**) HUVEC/vector pH 6.4, (**C**) HUVEC/GPR4 pH 7.4, and (**D**) HUVEC/GPR4 pH 6.4. HUVECs were treated with pH stimulation medium for 5 hours. Scale bar indicates 100µm. Arrows indicate endothelial cell borders with reduced VE-Cadherin protein expression.

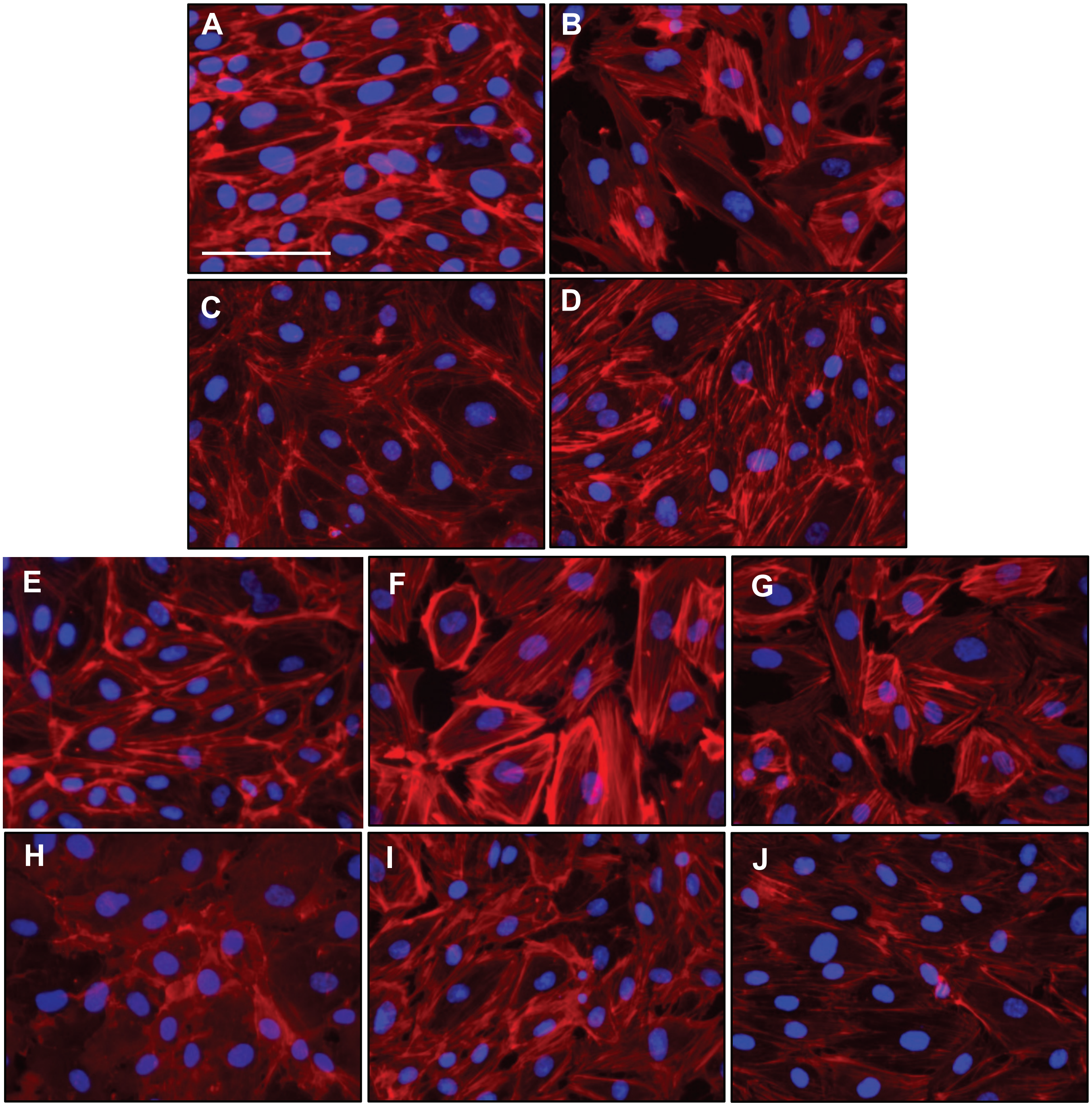

Figure S4. Inhibition of the $G_{\alpha12/13}$/Rho GTPase/ROCK/MLCK/actin cytoskeleton pathway decreases acidosis/GPR4-induced actin stress fiber formation in HUVECs. Related to Figure 3. (**A-J**) Actin stress fiber formation in HUVEC/GPR4 cells treated with various inhibitors, including p115-RGS (an inhibitory construct of $G_{\alpha12/13}$), thiazovivin (a Rho-associated kinase ROCK inhibitor), staurosporine (a myosin light chain kinase MLCK inhibitor), and cytochalasin D (an actin cytoskeleton inhibitor). Representative images of F-actin labeled with rhodamine phalloidin in (**A**) HUVEC/GPR4/pQCXIP vector pH 7.4, (**B**) HUVEC/GPR4/pQCXIP vector pH 6.4, (**C**) HUVEC/GPR4/p115-RGS pH 7.4, (**D**) HUVEC/GPR4/p115-RGS pH 6.4, (**E**) HUVEC/GPR4 pH 7.4, (**F**) HUVEC/GPR4 pH 6.4, (**G**) HUVEC/GPR4 pH 6.4 DMSO, (**H**) HUVEC/GPR4 pH 6.4 thiazovivin, (**I**) HUVEC/GPR4 pH 6.4 staurosporine, and (**J**) HUVEC/GPR4 pH 6.4 cytochalasin D. HUVECs were treated with pH stimulation medium for 5 hours. Scale bar indicates 100µm.

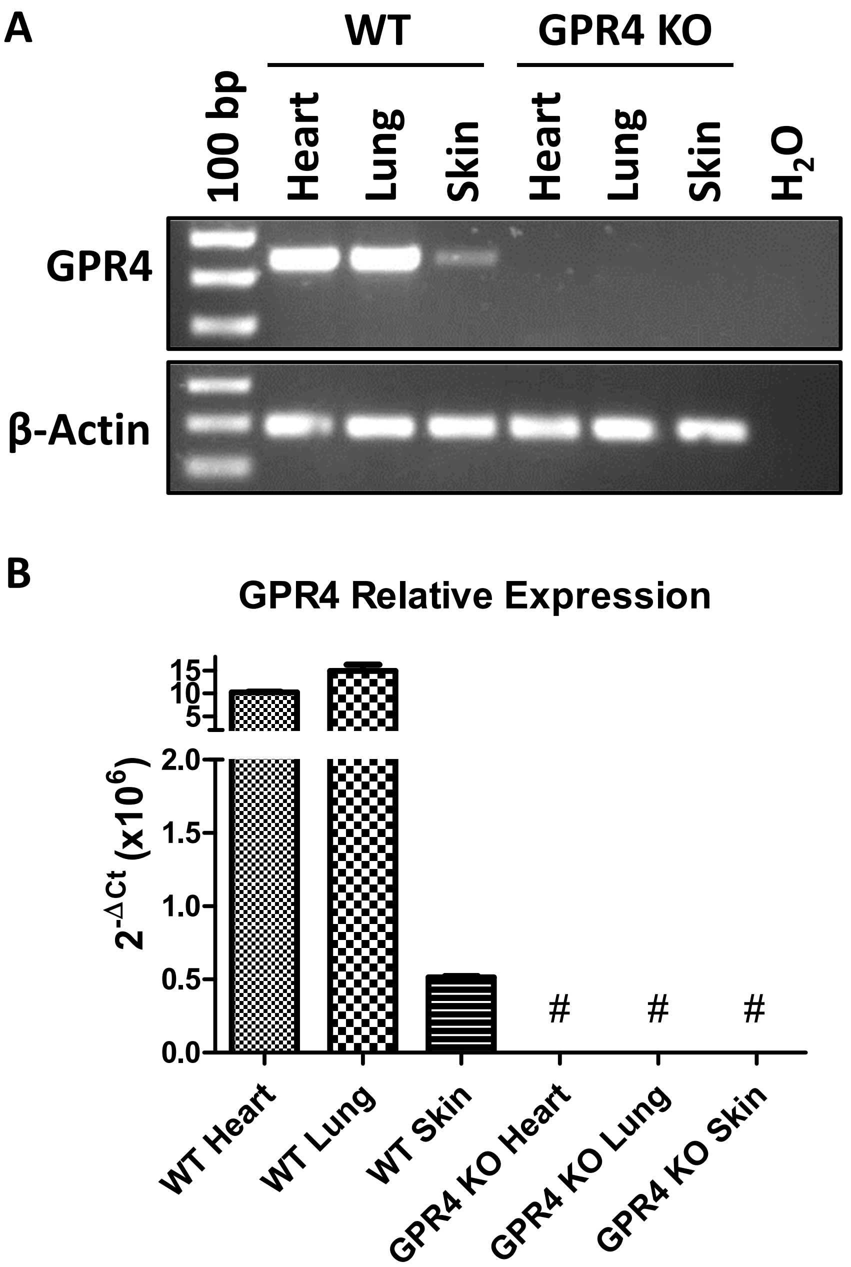

Figure S5. Absence of GPR4 mRNA expression in the GPR4 knockout mouse tissues. Related to Figures 5, 6 and 7. (**A**) RT-PCR and (**B**) real-time RT-PCR were performed to assess the expression of GPR4 mRNA in wild-type (WT) and GPR4 knockout (KO) mouse tissues. GPR4 mRNA expression was detected in the WT mouse heart, lung, and skin tissues but was absent in the GPR4 KO mouse tissues. Each cDNA sample was run in duplicate for the real-time RT-PCR analysis, and the 18S rRNA was used as the internal control for data normalization. The $2^{-\Delta Ct}$ method was used for data analysis. # not detectable.

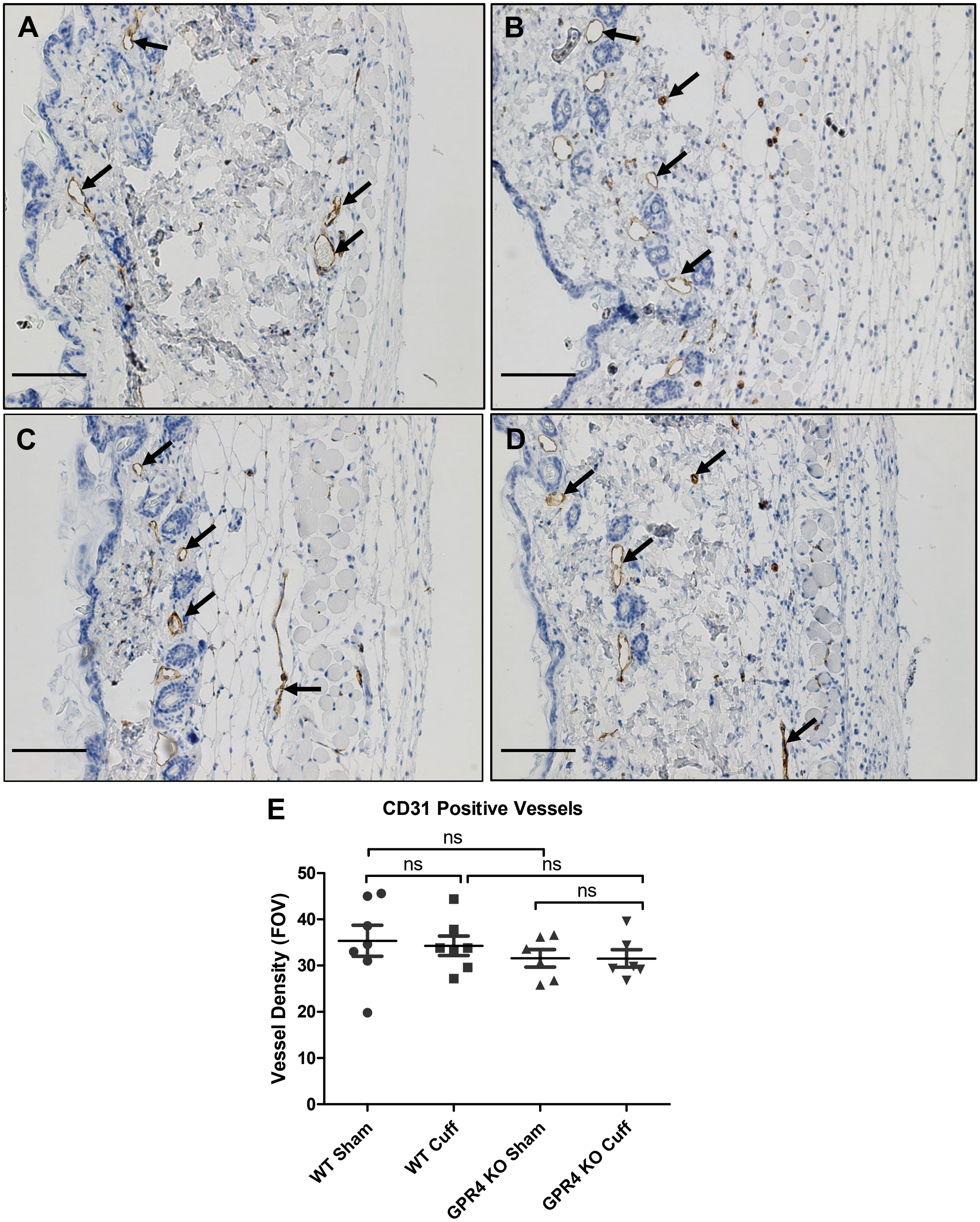

Figure S6. Blood vessel density in WT and GPR4 KO mouse hindlimb dermal tissues. Related to Figure 6. (**A-E**) Blood vessels were delineated by immunohistochemistry (IHC) with a pan-endothelial cell marker CD31 in the sham or cuff-affected mouse hindlimb dermal tissues. (**A**) WT sham, (**B**) WT cuff, (**C**) GPR4 KO sham, (**D**) GPR4 KO cuff, and (**E**) blood vessel density per field of view (FOV). No significant difference in blood vessel density was observed between WT and GPR4 KO or between sham and cuff affected dermal tissues. Arrows indicate blood vessels. 20x microscope objectives used. Scale bars indicate 100µm. Data are presented as mean ± SEM and was analyzed for statistical significance using the ANOVA. ns: not significant.

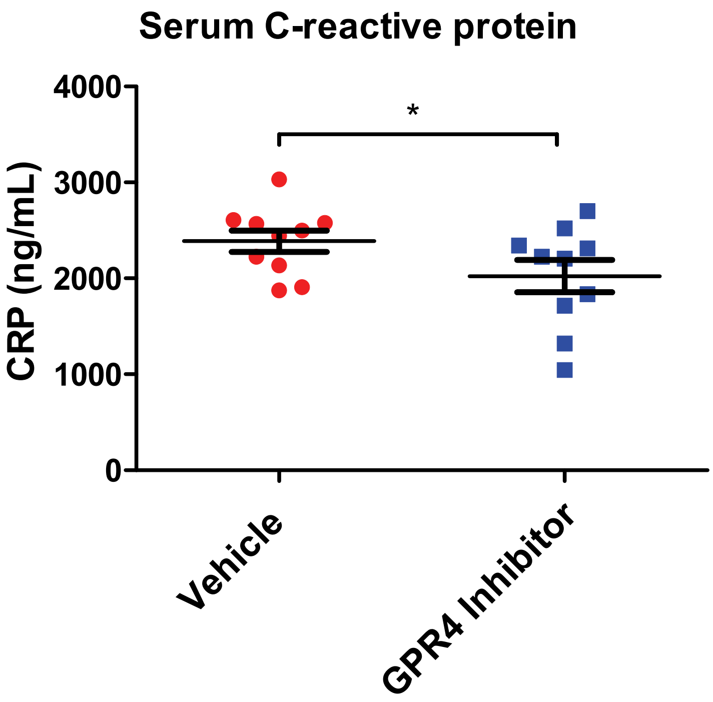

Figure S7. Mice treated with GPR4 antagonist 13 have reduced serum C-reactive protein levels when compared to vehicle control. Related to Figure 9. C-reactive protein (CRP) inflammatory marker in mouse serum. As previous indicators suggest GPR4 antagonist 13 reduces inflammation in the hindlimb ischemia-reperfusion mouse model, we assessed the anti-inflammatory effects of GPR4 antagonist 13 in the reduction of CRP levels in mouse serum. Mouse serum CRP was measured by ELISA and represented as ng/mL. Data analyzed from 10 WT/vehicle and 10 WT/GPR4 antagonist 13 mice. Data are presented as mean ± SEM and analyzed for statistical significance using the unpaired one-tailed *t*-test where $*p<0.05$.

Transparent Methods

Chemicals and reagents. For endothelial cell monolayer verification and live-cell staining, CellMask Deep Red Plasma Membrane Stain (#C10046) and NucBlue Live ReadyProbes Reagent (#R37605) were purchased from ThermoFisher Scientific (Waltham, MA, USA). The monoclonal antibody for VE-Cadherin (D87F2) was purchased from Cell Signaling Technology (Danvers, MA, USA). Secondary antibody, Rhodamine Red-X goat anti-rabbit (ThermoFisher Scientific) was used for all immunofluorescence experiments. Cellular permeabilization agent Triton X-100 detergent was purchased from BioRad (Hercules, CA, USA). Rhodamine Phalloidin used for F-actin florescence staining was purchased from Cytoskeleton (Denver, CO, USA). For immunofluorescence staining, we used Vectashield Antifade Mounting Medium with DAPI from Vector Laboratories (Burlingame, CA, USA). To evaluate *in vitro* endothelial cell permeability, fluorescein isothiocyanate (FITC)-dextran (average molecular weight 10,000 Daltons) was purchased from Millipore Sigma (St. Louis, MO, USA). The chemicals used for buffering the pH stimulation media include 4-(2-hydroxyethyl)-1-piperazineethanesulfonic acid (HEPES), N-(2-hydroxyethyl)-piperazine-N′-3-propanesulfonic acid (EPPS), 2-(4-morpholino)-ethanesulfonic acid (MES) and were purchased from Sigma-Aldrich (St Louis, MO, USA).

The GPR4 inhibitor, 2-Ethyl-3-(4-(E-3-(4-isopropyl-piperazin-1-yl)-propenyl)-benzyl)-5,7-dimethyl-3H-imidazo(4,5-b)pyridine (abbreviated as EIDIP), was purchased from Dalton Pharma Services (Toronto, ON, Canada) as previously described (Dong et al., 2017; Dong et al., 2013). The new generation of GPR4 inhibitor, 2-(4-((2-Ethyl-5,7-dimethylpyrazolo[1,5-a]pyrimidin-3-yl)methyl)phenyl)-5-(piperidin-4-yl)-1,3,4-oxadiazole (GPR4 antagonist 13, also known as NE-52-QQ57), was provided by Novartis Pharmaceuticals (Basel, Switzerland). The

vehicle for GPR4 antagonist 13 was a combination of 0.5% methylcellulose purchased from BioConcept AG (Allschwil, Switzerland) and 0.5% Tween-80 purchased from Sigma-Aldrich (St. Louis, MO, USA) and 99% water.

Cell culture. Primary human umbilical vein endothelial cells (HUVEC), human pulmonary artery endothelial cells (HPAEC), and human lung microvascular endothelial cells (HMVEC-Lung) (Lonza, Walkersville, MD, USA), and primary human colon microvascular endothelial cells (HMVEC-Colon) (Cell Biologics Inc., Chicago, IL, USA) were cultured at 37°C and 5% CO_2 in a humidified incubator for experimentation. HUVEC and HPAEC were cultured in endothelial cell growth medium 2 (EGM-2), while HMVEC-Lung and HMVEC-Colon were grown in EGM-2-MV medium (Lonza, Walkersville, MD, USA).

HUVECs stably expressing MSCV-IRES-GFP (HUVEC/Vector), MSCV-huGPR4-IRES-GFP (HUVEC/GPR4), MSCV-huGPR4 R115A-IRES-GFP (HUVEC/GPR4-R115A), Flink-control shRNA (HUVEC/Control shRNA), or Flink-huGPR4 shRNA (HUVEC/GPR4 shRNA) were developed by retroviral transduction and sorted by fluorescence-activated cell sorting (FACS) as previously described (Chen et al., 2011; Dong et al., 2017; Dong et al., 2013). To generate $G\alpha_{12/13}$ signaling deficient HUVECs, the p115 RGS construct was cloned into the pQCXIP vector as previously described (Kozasa et al., 1998; Yang et al., 2005). The p115 RGS-pQCXIP and empty pQCXIP retroviral vectors were stably transduced into the HUVEC/vector and HUVEC/GPR4 cells which were selected with 2µg/mL puromycin for 48 hours. Cells were used within 8 passages for all experiments.

Gap formation assay. Endothelial cells were seeded at $1x10^5$ cells/well in a 24-well tissue culture treated plate and were cultured to a confluent monolayer. Intact cellular monolayers were confirmed using a deep red cellular membrane dye (ThermoFisher, #C10046, Waltham, MA, USA) and a live cell nuclear dye (ThermoFisher, #R37605, Waltham, MA, USA). Endothelial cells were then treated with cell culture medium buffered to physiological pH 7.4 or acidic pH 6.4 for defined time points as previously described (Chen et al., 2011; Dong et al., 2017; Dong et al., 2013). For assessment of pharmacological inhibition of GPR4 *in vitro*, the GPR4 inhibitor (EIDIP, Dalton Pharma) was added to the pH stimulation media following the GPR4 inhibitor pretreatment for 1 hour in physiological pH 7.4 (Dong et al., 2017; Dong et al., 2013). Pictures of endothelial cell monolayers were obtained hourly over a 5-hour period using the EVOS Digital Inverted Microscope imaging system. ImageJ software (NIH) was used to quantify gap percentage per field of view (FOV).

Fluorescent filamentous-actin stress fiber formation assessment. Following the gap formation assay, endothelial cells were washed with DPBS and fixed with 4% paraformaldehyde for 15 minutes. Cells were then permeabilized for 10 minutes with 0.1% Triton X-100 and stained with Rhodamine Phalloidin for 30 minutes (Cytoskeleton Inc., Denver, CO, USA). Cells were then washed in DPBS and mounted with DAPI-containing Vectashield mounting media (Vector Laboratories, H-1200, Burlingame, CA, USA). Cells were imaged using the EVOS Digital Inverted Microscope imaging system.

Immunofluorescence. The endothelial cell monolayers in 24-well plates were treated with physiological pH 7.4 or acidic pH 6.4 media for 5 hours. Following pH treatments, cells were fixed

with 4% paraformaldehyde for 15 minutes, permeabilized with 0.1% Triton X-100 for 5 minutes and blocked with goat serum in phosphate buffered saline with 0.1% Tween-20 for 1 hour. The cells were then incubated with VE-cadherin primary antibody (Cell Signaling Technology, D87F2) in the blocking solution overnight at 4°C. Cells were washed and incubated for 1 hour with Rhodamine Red-X goat anti-rabbit secondary antibody. The wells were then washed five times with PBS and mounted with a glass cover slip using Vectashield with DAPI. The stained cells were viewed and pictures taken with an EVOS Digital Inverted Microscope.

Cellular permeability assay. Endothelial cell monolayers were cultured to confluency on a Transwell membrane insert with 0.4µm pores (Corning, NY, USA) within a 24-well plate and subsequently treated with physiological pH 7.4 or acidic pH 6.4 media for 5 hours. Following pH treatment, fluorescein isothiocyanate (FITC)-dextran with average molecular weight of 10,000 daltons (Millipore Sigma, St. Louis, MO, USA) diluted into pH stimulation medium was added into the upper chamber of the Transwell insert at a concentration of 1mg/mL. Subsequently, 1 mL of endothelial growth medium was also added to the lower chamber and the Transwell was incubated for 30 minutes at 5% CO_2 at 37°C. Following the incubation, 100µL of medium was drawn from the lower chamber and the FITC-dextran signal was quantified with a microplate reader at excitation/emission wavelength of 490/520 nm. The degree of cell permeability was determined by comparing the FITC-dextran fluorescent signal from the treatment group to the control group.

Tourniquet-based acute hindlimb ischemia and reperfusion murine model. All animal experiments were performed on 9-13-week old male and female mice. GPR4 wild-type (WT) or

GPR4-deficient (GPR4 KO) mice backcrossed into the C57BL/6 genetic background for 11 generations were used (Yang et al., 2007). To assess the inflammatory response post ischemia-reperfusion, the mouse hindlimb ischemia reperfusion injury model was employed using the pneumatic digital tourniquet cuff system (Model DC1.6, Hokanson, Inc., Bellevue, WA, USA). Prior to tourniquet application, a single dose of 0.03 mg/kg Buprenorphine analgesics was administered subcutaneously to each mouse. Mice were then anesthetized with 1% isoflurane inhalation and placed on a temperature-controlled pad followed by the tourniquet application to the left proximal hindlimb under 200 mm Hg regulated pressure for 3hrs as previously described (Bonheur et al., 2004). During the tourniquet-induced hindlimb ischemia procedure, the mouse breathing rate and body temperature was monitored. Mice were injected subcutaneously with 0.5 mL warm saline at the 1.5 hour and 3 hour time points for maintenance of hydration. After the tourniquet was released, the mice were placed into the microisolation cages with soft bedding for a 21 hour hindlimb reperfusion. Hindlimb circumference was measured before the procedure (baseline) and after the ischemia-reperfusion (IR), and the change in hindlimb circumference (value after IR – value at baseline) was used as an indicator to assess tissue edema. For the evaluation of GPR4 antagonist 13 (also known as NE-52-QQ57, Novartis) in the ischemia-reperfusion mouse model, both C57BL/6J and GPR4 WT mice were used for experiments ranging from 9 to 13 weeks old. Mice were administered vehicle control (0.5% methylcellulose/ 0.5% Tween 80/ 99% water) or GPR4 antagonist 13 at 30 mg/kg b.i.d. by oral gavage one day prior to experimental procedure. Mice were given a second dose of vehicle or GPR4 antagonist 13 approximately 2 hours prior to tourniquet cuff procedure. The last dose of either vehicle or GPR4 antagonist 13 was administered 6 hours into the reperfusion phase. The dosage of 30 mg/kg b.i.d. was chosen because of the best efficacy observed in a previous study (Velcicky et al., 2017). All

animal experiments were approved by the Institutional Animal Care & Use Committee of East Carolina University and were in accordance with the *Guide for the Care and Use of Laboratory Animals* administered by the Office of Laboratory Animal Welfare, NIH.

Tissue collection and histological analysis. After the 24 hour hindlimb ischemia-reperfusion procedure, mice were euthanized and the lower body was dissected for tissue collection. Upon dissection, inflammatory exudate within the interstitial space between upper leg muscle fibrous sheath and skin from the tourniquet-affected hindlimb was weighed and collected. A portion of the hindlimb skin containing inflammatory exudate was removed approximately 20 x 6 mm and fixed in 10% buffered formalin for tissue processing. The remaining exudate on the skin of the mouse was dissected and weighed. Tissues were embedded in paraffin, sectioned at 7µm, and stained with hematoxylin and eosin (H&E) for histopathological analyses. For assessment of neutrophilic infiltration into inflammatory exudate, pictures were taken from H&E tissue sections at five random points with a 20x objective. Leukocytes with the distinct polymorphic nuclear morphology were counted in each field of view (FOV) using ImageJ software in a blind manner as previously described (Sanderlin et al., 2017). Pictures were taken using the Zeiss Axio Imager A1 microscope.

Immunohistochemistry. Immunohistochemistry (IHC) was performed on serial sections of 7µm paraffin embedded skin with inflammatory exudate. Tissues were deparaffinized and hydrated to water by a series of declining ethanol concentrations. Antigen retrieval was performed using Tris-EDTA (pH 9.0) with 0.1% Tween 20 to assess protein expression using the SuperPicture 3rd Gen Immunohistochemistry detection system (Invitrogen, Waltham, MA). Endogenous peroxidases

were quenched and endogenous mouse IgG in blood serum was blocked using the Mouse-on-Mouse blocking reagent (Vector Laboratories, Burlingame, CA). Subsequently, 10% normal goat serum was used to block tissue followed by primary antibody incubation with anti-VCAM-1 (Abcam, ab134047), E-selectin (Abcam, ab18981, Cambridge, MA), and CD31 (Cell Signaling Technology, #77699, Danvers, MA) overnight at 4 °C and followed by incubation with secondary antibody. For detection, the Rabbit Vectastain Elite ABC-HRP Kit (Vector Laboratories, Burlingame, CA, USA) was employed according to the manufacture's protocol. After IHC was performed, pictures were taken using a Zeiss Axio Imager A1 microscope. VCAM-1 and E-selectin intensity in vascular endothelial cells were assessed using the scoring criteria as previously described: 1 = none/minimal, 2 = mild, 3 = moderate, 4 = high signal intensity (Sanderlin et al., 2019). To quantify blood vessel density, CD31-positive blood vessels per field of view (FOV) was counted.

To assess mouse plasma IgG protein in the exudate, the mouse Vectastain Elite ABC-HRP Kit was used (Vector Laboratories, Burlingame, CA). Endogenous peroxidase was quenched, blocking serum and biotinylated anti-mouse IgG secondary antibody was used followed by ImmPACT DAB Peroxidase Substrate (Vector Laboratories, Burlingame, CA) to visualize IgG leakiness from plasma. Immunohistochemical staining of tissue sections with no anti-mouse IgG antibody and the use of anti-Rabbit IgG antibody (Vector Laboratories, Burlingame, CA) were used for negative control. Pictures were taken with a Zeiss Axio Imager A1 microscope.

Enzyme-linked immunosorbent assay (ELISA). ELISA was performed to measure the C-reactive protein (CRP) level in the serum of mice treated with the GPR4 antagonist 13 or vehicle control. Immediately after the mice were euthanized, blood was collected through cardiac puncture

and allowed to form clots. After centrifugation, mouse serum on top of the clots was collected and stored in a -80 ^{o}C freezer for further analysis. The CRP concentration in mouse serum was measured using the mouse CRP ELISA kit (Sigma-Aldrich, RAB1121) according to the manufacturer's protocol.

RNA isolation and real-time RT-PCR. Total RNA was isolated from HUVECs and mouse tissues using the DNA/RNA/Protein extraction kit (IBI Scientific, Peosta, IA) and reverse transcribed into cDNA using the SuperScript IV reverse transcriptase (ThermoFisher Scientific, Waltham, MA). Real-time PCR was performed in duplicate with a program of 50°C for 2 min, 95°C for 10 min followed by 40 cycles of 95°C for 15 sec and 60°C for 1 min. Data was acquired using the QuantStudio 3 Real-Time PCR system (ThermoFisher Scientific, Waltham, MA) and analyzed using the $2^{-\Delta Ct}$ method. TaqMan primer-probe sets used for gene expression analysis include 18S rRNA (Hs99999901_s1), mouse Gpr4 (Mm00558777_s1), and human GPR4 (custom designed) as previously described (Chen et al., 2011; Dong et al., 2013). Additionally, regular RT-PCR was performed to assess mouse Gpr4 expression in WT and GPR4 KO mouse tissues as previously described (Yang et al., 2007). The mouse Gpr4 primer sequences for regular RT-PCR were: (sense) 5'-CAAGACCCACTTGGACCACA-3' and (antisense) 5'-TGTCCTGGGCCTCCTTTCTA-3'.

Statistical Analysis. GraphPad Prism software was used for statistical analysis. The results were recorded as the mean ± standard error from at least three independent experiments. For three or more groups, ANOVA was used followed by Bonferroni post hoc test. Statistical analyses between two groups were performed using the *t*-test. $P < 0.05$ was considered statistically significant.

Supplemental References

Bonheur, J.A., Albadawi, H., Patton, G.M., and Watkins, M.T. (2004). A noninvasive murine model of hind limb ischemia-reperfusion injury. J Surg Res *116*, 55-63.

Chen, A., Dong, L., Leffler, N.R., Asch, A.S., Witte, O.N., and Yang, L.V. (2011). Activation of GPR4 by acidosis increases endothelial cell adhesion through the cAMP/Epac pathway. PLoS One *6*, e27586.

Dong, L., Krewson, E.A., and Yang, L.V. (2017). Acidosis Activates Endoplasmic Reticulum Stress Pathways through GPR4 in Human Vascular Endothelial Cells. Int J Mol Sci *18*.

Dong, L., Li, Z., Leffler, N.R., Asch, A.S., Chi, J.T., and Yang, L.V. (2013). Acidosis Activation of the Proton-Sensing GPR4 Receptor Stimulates Vascular Endothelial Cell Inflammatory Responses Revealed by Transcriptome Analysis. PLoS One *8*, e61991.

Kozasa, T., Jiang, X., Hart, M.J., Sternweis, P.M., Singer, W.D., Gilman, A.G., Bollag, G., and Sternweis, P.C. (1998). p115 RhoGEF, a GTPase activating protein for Galpha12 and Galpha13. Science *280*, 2109-2111.

Sanderlin, E.J., Leffler, N.R., Lertpiriyapong, K., Cai, Q., Hong, H., Bakthavatchalu, V., Fox, J.G., Oswald, J.Z., Justus, C.R., Krewson, E.A., *et al.* (2017). GPR4 deficiency alleviates intestinal inflammation in a mouse model of acute experimental colitis. Biochim Biophys Acta *1863*, 569-584.

Sanderlin, E.J., Marie, M., Velcicky, J., Loetscher, P., and Yang, L.V. (2019). Pharmacological inhibition of GPR4 remediates intestinal inflammation in a mouse colitis model. Eur J Pharmacol *852*, 218-230.

Velcicky, J., Miltz, W., Oberhauser, B., Orain, D., Vaupel, A., Weigand, K., Dawson King, J., Littlewood-Evans, A., Nash, M., Feifel, R., *et al.* (2017). Development of Selective, Orally Active GPR4 Antagonists with Modulatory Effects on Nociception, Inflammation, and Angiogenesis. J Med Chem *60*, 3672-3683.

Yang, L.V., Radu, C.G., Roy, M., Lee, S., McLaughlin, J., Teitell, M.A., Iruela-Arispe, M.L., and Witte, O.N. (2007). Vascular abnormalities in mice deficient for the G protein-coupled receptor GPR4 that functions as a pH sensor. Mol Cell Biol *27*, 1334-1347.

Yang, L.V., Radu, C.G., Wang, L., Riedinger, M., and Witte, O.N. (2005). Gi-independent macrophage chemotaxis to lysophosphatidylcholine via the immunoregulatory GPCR G2A. Blood *105*, 1127-1134.
